# The impact of expectancy on cognitive performance during alcohol hangover

**DOI:** 10.1186/s13104-018-3827-2

**Published:** 2018-10-18

**Authors:** Lydia E. Devenney, Kieran B. Coyle, Joris C. Verster

**Affiliations:** 1 Ulster University, Derry, Northern Ireland, UK; 2 Division of Pharmacology, Utrecht University, Universiteitsweg 99, 3584 CG Utrecht, The Netherlands; 3 Institute for Risk Assessment Sciences (IRAS), Utrecht University, Utrecht, The Netherlands; 4 Centre for Human Psychopharmacology, Swinburne University, Melbourne, Australia

**Keywords:** Alcohol, Hangover, Expectancy, Blinding

## Abstract

**Objective:**

Knowing the purpose of a clinical study may provoke expectancies among subjects that may influence the study outcome. For example, expectancies about a drug effect may cause subjects to put in more effort to counteract these effects on performance tasks, or cause stress or other mood alterations in anticipation of expected adverse effects. The objective of this study was to investigate to what extent expectancy effects will influence the magnitude of cognitive performance decrement in the alcohol hangover state.

**Results:**

Forty subjects with a mean (SD) age of 24.0 (7.4) years old participated in a naturalistic study to examine the alcohol hangover effects on cognitive performance. Subjects in the expectancy group were informed of the purpose of the study. In the control group subjects were told that the purpose of the study was to investigate the effects of time of day on cognitive performance. Subjects consumed a mean (SD) of 12.9 (10.0) alcoholic drinks the night before testing. Cognitive tests included the Stroop test, Eriksen’s flanker test, a divided attention test, intra-extra dimensional set shifting test, spatial working memory test, and free word recall test. Expectancy effects did not differentially affect cognitive performance in the alcohol hangover state.

## Introduction

Subjects in clinical trials can have expectancies with regards to possible drug effects on performance. Also, knowledge about possible adverse effects may affect the study outcome. These expectancy effects are seen with commonly used substances such as caffeine and alcohol [1, 2], but expectancies can also be present when subjects are drug-naïve. The latter can for example happen if subjects of a clinical trial are informed about the potential effects of a newly developed drug. If a drug under investigation is told to negatively affect performance, subjects may put more effort to counteract this expected effect [3, 4]. In addition, they may adapt their test performance. For example, subjects may be more cautious than usual on a driving test and chose a lane position for the car which is closer to the road shoulder than they usually do [5]. Also, the risk of experiencing certain adverse effects (e.g., heart racing) may cause fear or distress among study subjects which can influence test performance [6]. These behavioral adaptations are often subtle and both subjects and investigators are not always aware of their presence or impact on their behavior.

To overcome the influence of these expectancies studies are usually single blind or double blind, and compare the drug with a placebo that has the same appearance and taste of the drug under investigation. In a single blind design the subjects are unaware when they receive which treatment, in a double blind design the researchers are also unaware which treatments they administer. A double-blind design is preferred above a single blind design, as the researchers can also have expectancies about the drug effects. Expectancies of researchers can unintendedly influence subjects behavior and mood, for example by treating subjects differently in the drug and placebo condition (e.g., being more cautious due to expected adverse effects, or paying more attention to giving test instructions to subjects who are potentially sedated by a drug).

After blinding and randomization of the treatment order, the subject (and/or investigator) is unaware on which test day they receive a drug or placebo treatment. Thus, adequate blinding ensures that subjects do not know when they received a drug or a placebo treatment. This does however not imply concealing the drugs that are administered and/or the purpose of a study [7]: subjects may still have expectancies with regard to the effects of a drug, and if blinding is incomplete this may still affect the study outcome.

Unfortunately, blinding is not always successful [8]. For example, blinding is an issue of debate in alcohol hangover research [9, 10]. The alcohol hangover refers to the combination of mental and physical symptoms, experienced the day after a single episode of heavy drinking, starting when blood alcohol concentration approaches zero [11]. Given the familiarity of drinkers with alcohol intoxication effects, and the relative high amounts of alcohol required to provoke a hangover, proper blinding seems impossible.

Literature reviews have raised concerns about expectancy effects in naturalistic studies examining the alcohol hangover state [10]. In naturalistic studies, blinding is absent as subjects consume alcohol on their own, without interference of the researchers, and address at the Institute the following morning to be tested in the hangover state. To date, investigations of expectancy have not been carried out on hungover subjects. It is important to examine whether expectancy plays a role in naturalistic hangover studies. If this is the case, methodologies may need to be adapted to reduce expectancy, for example by concealing the purpose of the study. The aim of the current study was therefore to examine whether expectancy about the purpose of a naturalistic hangover study has an impact on cognitive impairment after an evening of alcohol consumption.

## Main text

### Methods

#### Subjects and design

N = 40 healthy social drinkers (students) were recruited at Ulster University Halls of Residence. The study had a naturalistic design [12]. Subjects were recruited in the morning *after* the drinking session (which was not part of the study), and directly after signing informed consent they participated in the study. Thus, there was no intervention and subjects consumed alcohol on their own without interference of the researchers. Advantage was taken of the predictability of student drinking with regards to recruitment. Tuesdays and Thursday were popular student nights at the time of recruitment, therefore, Wednesday and Friday mornings were used to collect data. Testing took place between 9 and 11.30 a.m. Subjects were included if the consumed alcohol the evening before. Subjects with a BAC level above zero at the time of recruitment were excluded from the study.

During recruitment, subjects were allocated to one of two groups. The first group was told they were participating in a study examining their cognitive performance during alcohol hangover. In the second group alcohol hangover was not mentioned. Instead, they were informed that the purpose of the study was to investigate the effects of time of day on cognitive performance.

#### Procedure

After arriving at the Institute, subjects completed the Acute Hangover Scale [13], demographic information was collected, and information was gathered about the number of hours sleep and previous night’s alcohol consumption. Thereafter, a cognitive test battery was completed.

Demographics are summarized in Table 1. Five participants did not report hangover symptoms and had an AHS score of zero. Therefore all analyses were run twice: first including all subject, and second including only those subjects who reported having a hangover. For both analysis, no significant differences were observed between the expectancy group and the control group in alcohol consumption variables and hangover severity.

**Table 1:** Demographics and alcohol consumption variables of the expectancy group and control group

	All subjects				AHS > 0 only			
	Expectancy group	Control group	Total	p-value	Expectancy group	Control group	Total	p-value
N	20	20	40		17	18	35	
Male/female	8/12	13/7	21/19	.12	7/10	12/6	19.16	.14
Age	23.7 (7.9)	24.2 (7.1)	24.0 (7.4)	.83	21.2 (3.0)	23.6 (6.7)	22.4 (5.3)	.18
Age of first drink	16.6 (7.1)	15.4 (1.5)	16.0 (5.1)	.48	15.1 (1.2)	15.4 (1.5)	15.3 (1.4)	.57
Alcoholic drinks on night out	15.1 (11.0)	10.6 (8.6)	12.9 (10.0)	.16	14.9 (12.4)	11.4 (8.6)	12.7 (10.5)	.48
Hours of sleep	6.0 (2.2)	6.4 (1.7)	6.2 (1.9)	.55	6.1 (2.2)	5.9 (2.3)	6.0 (2.2)	.75
Alcohol hangover severity	14.6 (12.7)	10.4 (10.6)	12.5 (11.7)	.26	17.1 (12.1)	11.5 (10.5)	14.2 (11.5)	.15

Alcohol hangover severity was assesses with the Acute Hangover Scale [13]

Significant differences (p < .05) are indicated by *

#### Tasks

*Eriksen’s Flanker task* In this selective spatial attention task the targets and distracters consist of the letters A and B [14]. Distracters are presented at either side of the target and appear either near (1 cm) or far (3.4 cm) from the target. Distracters are either compatible (AAA) or incompatible with the target (BAB). Subjects are required to respond to the target letter by pressing an appropriate key as quickly and accurately as possible. Outcome variables include ‘total errors’, ‘distance’ and ‘compatibility’ response times. Distance is calculated by subtracting response times (RTs) to far items from near items, and compatibility is computed by subtracting compatible items from incompatible items.

*Stroop* In this task, words are presented on the screen one at a time in Blue, Green, Red, Purple and Brown as used in the original task [15, 16]. Ignoring the text-meaning of the words, subjects are required to respond to the font color only by using the corresponding buttons on the keyboard provided. Outcome variables include the number of errors and Stroop interference. Stroop interference represents the difference between RTs for congruent (e.g. red presented in red font) and incongruent items (e.g. red presented in green font).

*Divided attention test* In this test [17, 18], a series of single digits appear in the center of a computer screen at a rate of one per second. When three consecutive odd numbers appeared (e.g. 3, 5, 7) in the center of the screen subjects are required to respond appropriately using the keyboard in front of them (central, ‘Z’). Simultaneously, a blue box may appear left, right, below or above the center of the screen (peripheral). Subjects are required to respond when a blue box appears on the screen as quickly and accurately as possible by pressing ‘M’ on the keyboard. Outcome measures included total errors, central RTs and peripheral RTs.

*Free recall* The free recall task consists of twenty words that are presented on the computer screen. In the minute directly following presentation subjects are required to write down as many words as they can remember. The outcome measure is the number of correctly recalled words.

*Spatial working memory* The CANTAB spatial working memory task requires retention and manipulation of visuospatial information [19]. The subject must touch the colored squares in order to find a blue token. A number of colored boxes are shown on the screen, and the subject should find one yellow ‘token’ in each of a number of boxes and use them to fill up an empty column on the right-hand side of the screen [19]. Task difficulty varies as the number of boxes can be gradually increased and the color and position of the boxes changes from trial to trial to prevent predictability. The most efficient strategy is to choose an order to press the boxes, and start over in the same order each time a blue token is found. Outcome measures include number of errors (selecting boxes that have already been visited) and strategy. Higher strategy scores indicate poorer use of the best strategy.

*CANTAB*—*intra*-*extra dimensional set sifting* This test is a computerized analogue of the Wisconsin card sorting task which features visual discrimination and attentional set formation maintenance, shifting and flexibility of attention [19–21]. In this task, participants must use feedback to work out a rule that determines which stimulus is correct. After six correct responses, the stimuli and/or rule changes. Starting with simple stimuli (individually shown white lines and pink shapes) corresponding to intra-dimensional shifts in rules. Gradually, the task becomes more complex (e.g., white lines overlaid on the pink shapes) also requiring extra-dimensional rule shifting. Outcome measures are the number of intra- and extradimensional errors (i.e., failing to identify the strategy within 6 trials).

#### Statistical analysis

Statistical analysis were conducted with SPSS, version 24. Mean (SD) were computed for each variable. Results from the expectancy group and control group were compared using independent samples paired t-tests and considered significant if p < .05.

### Results

Table 2 summarizes the results from the cognitive test battery. No significant differences were found between the expectancy group and control group on any of the tests.

**Table 2:** Cognitive test performance

	All subjects			AHS > 0 only		
	Expectancy group	Control group	p-value	Expectancy group	Control group	p-value
*Stroop test*						
Mean RT—congruent items (ms)	1184.0 (188.5)	1300.5 (270.2)	.55	1193.8 (198.0)	1304.2 (273.8)	.18
Mean RT—incongruent items (ms)	1617.1 (343.0)	1680.7 (323.9)	.12	1649.2 (363.7)	1655.6 (329.1)	.96
Interference (ms)	433.2 (232.0)	380.2 (256.6)	.50	455.3 (242.0)	351.4 (219.8)	.19
Number of errors	5.5 (2.0)	5.5 (1.9)	1.00	5.5 (2.1)	5.5 (2.0)	.97
*Eriksen’s Flanker test*						
Compatibility RT (ms)	16.4 (60.1)	11.8 (35.6)	.77	15.7 (64.9)	11.8 (33.1)	.82
Distance RT (ms)	21.9 (32.7)	19.5 (50.6)	.86	23.0 (35.1)	17.5 (52.8)	.72
Number of errors	2.2 (1.4)	1.5 (1.7)	.16	2.20 (1.2)	1.6 (1.7)	.44
*Divided attention test*						
Mean RT—central stimuli (ms)	696.6 (190.7)	698.8 (178.0)	.97	702.6 (206.3)	671.3 (162.1)	.63
Mean RT—peripheral stimuli (ms)	774.0 (182.9)	768.8 (182.9)	.92	745.7 (178.6)	761.5 (111.6)	.76
Number of errors	3.4 (4.4)	3.1 (2.3)	.80	3.3 (4.3)	2.8 (2.3)	.70
*CANTAB*—*Intra*-*extra dimensional set shifting test*						
Extra-dimensional number of errors	13.9 (10.3)	11.4 (9.6)	.43	13.7 (10.7)	11.4 (9.9)	.52
Intra-dimensional number of errors	6.6 (2.5)	6.0 (4.3)	.56	6.1 (1.7)	6.0 (4.5)	.92
*CANTAB*—*spatial working memory test*						
Number of errors	21.6 (19.2)	22.5 (17.0)	.88	21.5 (18.1)	24.2 (17.2)	.67
Strategy score	30.3 (5.8)	28.3 (9.6)	.45	30.8 (5.7)	29.6 (6.6)	.58
*Free recall test*						
Number of correctly recalled words	8.2 (2.9)	6.9 (2.5)	.15	7.6 (2.7)	7.1 (2.5)	.55

*RT* response time, *AHS* Acute Hangover Scale

Significant differences (p < .05) are indicated by *

### Discussion

This study indicates that expectancies with regard to the purpose of a study have no significant impact on the study outcome. On none of the test outcomes a significant difference was observed between subjects for which the aims of the study were disclosed or concealed.

Our findings do not support previous concerns that expectancy is likely to significantly contaminate the results of studies using a naturalistic design [10]. The review by Stephens et al. provided no rationale as to why expectancy would influence the study outcome other than the argument that the advantage of concealing the study aim may help to mimic as close as possible “how hungover people behave in the real world” [10]. It can be questioned however whether this is correct. First, subjects are aware that they are participating in a research study thus may adapt their behavior accordingly, independent of the purpose of a study. Second, whether or not it is disclosed that the alcohol hangover is the topic under investigation, subjects will all be aware of the fact that they have consumed a large quantity of alcohol, and most of them will probably have the expectancy that the hangover effects will affect their test performance negatively. The impact of the latter may however depend on the level of tolerance drinkers have with regard to next-day alcohol effects, and the achieved peak BAC. It is unclear why Stephens et al. limited the possible impact of expectancies to naturalistic study designs. To our opinion, as blinding is imperfect in hangover research, expectancies can equally well impact controlled ‘double-blind’ studies. Due to unsubstantiated concerns of ethics committees with regard to the amount of alcohol that can be safely administered to subjects, the quantity of alcohol consumed in controlled trials is usually significantly lower when compared naturalistic studies in which ‘real life’ alcohol amounts are consumed. Notwithstanding this, also in single- or double-blind trials the target blood alcohol concentration (.08% or higher) is more than sufficient to unblind the alcohol condition.

Taken together, no significant expectancy effects were observed. The current data suggests that knowledge about the purpose of a research study did not differentially affect performance in the alcohol hangover state.

## Limitations

In this study, subjects with a positive BAC reading were excluded from participation. In total, these were 9 subjects. At the time of designing and conducting the study this was best practice as it was argued that a positive BAC could produce additional (acute) effects on performance [9]. However, current consensus [22] resulted in a definition of the alcohol hangover stating that hangovers occur when “…. BAC approaches zero” [11]. In retrospective it would have been both appropriate and interesting to also include subjects with a positive BAC reading in the study and determine to what extent this influences performance effects and may have an impact on expectancies.
